# Making cigarette taxes more effective in Mozambique: A simulation analysis using the Tobacco Excise Tax Simulation Model (TETSiM)

**DOI:** 10.1371/journal.pone.0341079

**Published:** 2026-02-02

**Authors:** Vanessa Darsamo, Zunda Chisha, Georgina Bonet Arroyo, Corné van Walbeek

**Affiliations:** 1 Research Unit on the Economics of Excisable Products, University of Cape Town, Cape Town, South Africa; 2 WHO FCTC Knowledge on Tobacco Taxation, University of Cape Town, Cape Town, South Africa; 3 World Health Organization, Maputo, Mozambique; Shandong University of Science and Technology, CHINA

## Abstract

Cigarette price tax shares in Mozambique are among the lowest in Southern Africa. The excise tax share of the price of the most-sold brand is 14.7%, and the total tax share is 28.5%. The average excise tax share for the Southern African Development Community is 36.7% and the average total tax share is 51.6%. We used the Tobacco Excise Tax Simulation Model (TETSiM), to simulate the impact of a substantial cigarette excise tax increase on prices, tax shares, consumption, tax revenue, and smoking prevalence, between 2023 and 2028. We simulated three scenarios. *Scenario 1* assumes that the cigarette excise tax adjustments are as stipulated in the Excise Tax on Specific Products Law for 2023–2025, and then increase by 4% annually between 2026 and 2028. *Scenario 2* proposes that the excise tax increases by the sum of inflation, income growth, and an additional 30%, annually. We assume full tax pass-through for both *scenarios 1* and *2*. In *scenario 3*, we use the same annual tax adjustments as *scenario 2*, but assume tax over-shifting for imported and most-sold brands. Our findings indicate that, by 2028, the excise tax share decreases from 14.7% to 13.9% in *scenario 1*, but increases to 40.2% in *scenario 2,* and to 35.8% in *scenario 3*. The total tax share drops from 28.5% to 27.7% in *scenario 1*, but increases to 53.9% in *scenario 2*, and to 48.1% in *scenario 3*. Smoking prevalence is expected to drop from 9.80% to 9.77% in *scenario 1,* to 8.23% in s*cenario* 2, and to 8.08% in *scenario 3*. *Scenario 2* results in the highest expected total tax revenues (173% increase). The study provides insights on the importance of appropriately revising cigarette tax policies in Mozambique. Substantial tax increases will contribute to a healthier population and a more sustainable fiscal landscape.

## Introduction

Tobacco use is decreasing in many countries globally, but continues to rise in many African countries [1]. Rapid population growth, a large proportion of young people, rapid economic growth (albeit from a low base), a desire to attract investment, weak institutions, and aggressive tobacco industry marketing all contribute to the rapid increase in tobacco consumption in Africa [2]. These forces apply to Mozambique as well.

Mozambique is a large country on the south-east coast of Africa. The country gained independence from Portuguese rule in 1975 [3]. Between 1976 and 1992, Mozambique had a civil war which destroyed much of the country’s infrastructure, caused about one million deaths, and displaced up to 6 million people [4,5]. Between the end of the civil war in 1992, and 2023, Mozambique’s population has grown from 13.8 million to 34 million [6]. Over this period, per-capita gross domestic product (GDP) grew at an average annual rate of 3.7%, from USD 203 in 1992 to USD 603 in 2023 (expressed in constant 2015 USD) [7]. Despite the fairly rapid economic growth, Mozambique has one of the lowest Human Development Indices (0.446), being ranked 185^th^ out of 191 countries in 2021 [8].

According to the STEPS survey, the prevalence of tobacco use was 18.7% in 2005 and dropped to 11.3% by 2014/2015 [9,10]. In 2020, the age-standardized use rate was estimated to be 13.2% for all tobacco products, smoked and smokeless tobacco, and 9.8% for cigarettes, among people aged 15 years and older [11]. Manufactured and hand-rolled cigarettes are the most common type of tobacco product in Mozambique. Smokeless tobacco is more popular among women than men [12].

Every year, tobacco use accounts for 3.5% (9,400) of all deaths in Mozambique and is a major risk factor for non-communicable diseases (NCDs), including cancer, diabetes, chronic respiratory diseases, and cardiovascular diseases [13]. It is estimated that reducing smoking rates, even moderately, or preventing rises in smoking rates, prevents thousands of cancer cases, as well as thousands of cases of other chronic diseases and premature deaths [14]. Tobacco use cost the country MZN 11.7 billion (USD 186 million), equivalent to 1.3% of the GDP, in 2019 [13].

Targeting NCD risk factors, such as tobacco use, through stronger tobacco-control measures is critical for preventing NCDs. Tax and price policies are widely recognized to be one of the most effective means of reducing the consumption of tobacco products [15]. Evidence indicates that increasing tobacco excise taxation reduces tobacco use while simultaneously increasing government tax revenue [16,17].

In order to curb tobacco use and the associated harms, the Mozambican government would do well to implement stringent taxation measures to increase the price of tobacco products and make them less affordable. This paper aims to simulate the impact of Mozambique’s approved tobacco excise-tax increases, as well as the likely impact of a more substantial annual excise-tax increase on cigarette prices, tax shares, cigarette consumption, the adult smoking prevalence rate and the cigarette tax revenue [18].

### Tobacco taxes in Mozambique

Tobacco taxes are regulated under the Excise Tax on Specific Products Law (ICE in Portuguese) [19]. Every three years, the Tax Authority proposes revisions and updates of the ICE taxes, including tobacco taxes. The proposal is submitted to the Ministry of Economy and Finance (MoF), who share it with all Government ministries for their input. The proposal is also shared with the Private Sector Association, the Chamber of Commerce, and the Tobacco Producer’s Association. The proposal is submitted to the Council of Ministries for their endorsement, and then to the Parliament for approval, and is then passed into law.

In 2009, cigarettes were taxed at 75% of the cost, insurance, and freight (CIF) value with a tax ceiling of MZN 6.00 (approximately USD 0.01), and a tax floor of MZN 2.40, per pack of 20 cigarettes [19]. The ICE was not revised until 2013. In January 2013 the *ad valorem* component was maintained, but the tax tiers were revised, covering the period 2013–2015. The most-sold brand was subject to the lower tax tier of MZN 3.80 per pack in 2013, MZN 4.90 per pack in 2014, and MZN 5.90 per pack in 2015. The higher tax tier was MZN 7.00 in 2013, MZN 7.15 in 2014, and MZN 8.00 per pack in 2015 [20]. In December 2017, the ICE was revised for the period 2018–2020. This ICE introduced a uniform specific excise tax on cigarettes, and abolished the *ad valorem* tax component. The nominal excise tax was set at MZN 8.00 in 2018, MZN 8.40 in 2019, and MZN 8.80 in 2020 [21].

The excise taxes were not revised in 2020 and 2021, due to the COVID-19 pandemic. The real, i.e., inflation-adjusted, excise tax per pack dropped by 9% between 2020 and 2022. In December 2022, the Mozambican government approved a new law covering the period 2023–2025. Under this new law, the excise tax increased by 14% (to MZN 10.00) in 2023 to account for the fact that there were no tax revisions between 2020 and 2022 [18]. The nominal excise tax rate is legislated to increase by 4.0% to MZN 10.40 in 2024 and by 3.9% to MZN 10.80 in 2025 [18].

**Fig 1:**
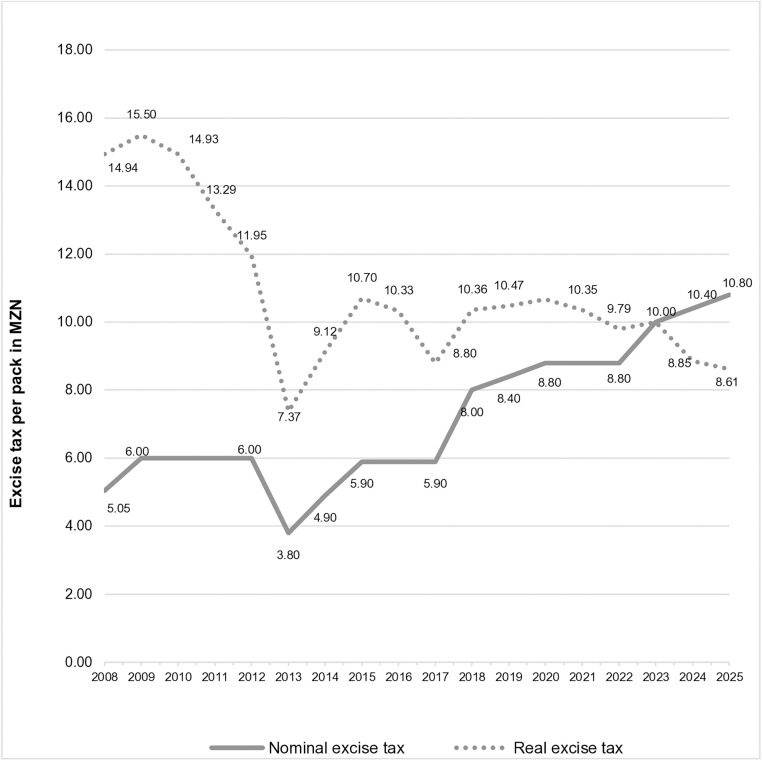
Nominal and real excise tax per cigarette pack of the most-sold brand*: actual (2008-2023), and legislated (2024–2025). * For 2008 to 2016, the graph only illustrates the minimum excise tax applicable to the most-sold brand. Source [6,18–24].

Between 2008 and 2023 the nominal excise tax increased by an average annual rate of 6.0%. However, these annual cigarette excise-tax increases were less than the inflation rate, which averaged 7.6% during that period [6]. In real terms, the excise tax decreased on average by 1.5% annually between 2008 and 2023. See Fig 1.

For the most-sold brand in Mozambique, the excise tax comprised only 14.7% of the retail price, while the total tax share (which includes the value-added tax or VAT) accounted for 28.5% of the retail price (MZN 60.00) in 2020. The average excise tax share and total tax share of the retail price of the most-sold brand was 36.7% and 51.6%, respectively, in 2020 for the 16-member Southern African Development Community (SADC) region [24]. Mozambique’s excise tax share and total tax share were the second lowest in the SADC region. Furthermore, in 2023, the Mozambican government reduced the VAT from 17% to 16% [25].

## Materials and methods

We constructed a Tobacco Excise Tax Simulation Model (TETSiM), based on the design by Van Walbeek [26] to simulate the impact of different cigarette excise-tax changes on the following variables: (1) cigarette prices, (2) cigarette tax shares, (3) cigarette consumption, (4) cigarette tax revenue, and (5) the adult cigarette-smoking prevalence rate, between 2023 and 2028. We use 2023 values as the baseline, and present our results in constant 2023 prices.

We divide the cigarette market in Mozambique into four price segments: imported, most-sold, economy, and illicit [23]. Based on information from the Mozambican customs authority, the illicit market accounts for about 5% of the total cigarette market [23].

Two crucial inputs for the TETSiM are the price elasticity and income elasticity of demand. The price elasticity of demand quantifies the relationship between a change in the real price and consumption. Based on a large international literature, we use different price elasticities for the various price segments (−0.5 for imported cigarettes, −0.6 for most-sold cigarettes, −0.7 for economy cigarettes, and −0.7 for illicit cigarettes) [15,16,27]. This implies that, on average, a 10% increase in the real price of cigarettes will decrease cigarette consumption by between 5% and 7%, holding all other factors constant. We use different demand elasticities for the different price segments as consumers’ responses to price and income changes vary. Consumers of premium brands, tend to be more affluent, and therefore less price sensitive than consumers of lower-priced brands [28–30].

Similarly, the income elasticity of demand measures the change in demand in response to a 1% change in income. International estimates of income elasticity of demand for legal tobacco products typically lie between zero and one, and most are smaller than 0.5 [15,31–34]. We assumed that the income elasticities are 0.3 for imported cigarettes, 0.4 for most-sold cigarettes and 0.5 for economy cigarettes [35]. Since illicit cigarettes are typically inferior and low-priced products, we used an income elasticity of −0.5 for them [36]. This means that, on average, a 10% increase in real per-capita GDP (the proxy for income that we use) is expected to increase legal cigarette demand by between 3% and 5%, and reduce the demand for illegal cigarettes by 5%, holding all other factors constant (see Table 1).

**Table 1:** TETSiM input data (all data refer to 2023).

Input variables	Imported	Most-sold	Economy	Illicit
Market share	2%	51%	42%	5%
Cigarette consumption (million packs)	2.15	54.75	45.09	5.37
Retail price per pack (current MZN)	155.00	68.00	40.00	20.00
Specific excise tax per pack (current MZN)	10.00	10.00	10.00	0.00
VAT rate	16%	16%	16%	0%
Income elasticity	0.30	0.40	0.50	−0.50
Price elasticity	−0.50	−0.60	−0.70	−0.70
Cross-price elasticity*	–	0.05	0.10	0.10

Source: [23].

*Refers to the the percentage increase in the quantity demanded of the cheaper segment in response to a 1% change in the price of the more expensive segment.

An increase in cigarette prices will reduce consumption in the individual market segments, but it could also change the *structure* of the market (in terms of the market segments). For example, if a brand becomes too expensive, some people will switch to cheaper brands. This switching behaviour is quantified by the cross-price elasticity of demand. In our model, the cross-price elasticity of demand is the percentage change in the consumption of cigarettes in the cheaper market segment in response to a 1% increase in the price of the cigarettes in the more expensive market segment. There is not a large empirical literature on cross-price elasticities for cigarettes, but the limited number of studies indicate that the cross-price elasticities are positive, highlighting that the cigarettes in the different price segments are substitutes [37–39]. We used cross-price elasticities that range from 0.05 to 0.10 (see Table 1).

The simulations are conducted for the period 2024–2028, with 2023 as the baseline. For real per capita GDP and inflation predictions, we use International Monetary Fund (IMF) numbers, and for population growth predictions, we use the Mozambican National Institute of Statistics (INS) numbers; see Table 2. The 2020 age-standardized current cigarette-smoking rate (9.8%) among people aged 15 years and older was used for our baseline (2023) [42].

**Table 2:** Input data for the TETSiM 2023–2028.

Period	Inflation rate (%)	Real GDP per capita growth rate (%)	Population growth rate (%)	CPI (calculated)
2023	4.30	3.50	2.50	1.00
2024	4.70	2.50	2.50	1.05
2025	5.50	2.50	2.50	1.10
2026	5.50	1.50	2.50	1.17
2027	5.50	10.60*	2.50	1.23
2028	5.50	9.60*	2.50	1.30

Sources: [6,40].

* The accelerated per capita GDP growth in 2027–2028 is attributed to the expected production of the liquefied natural gas by TotalEnergies [41].

### Simulation scenarios

We considered three possible tax scenarios. In the first scenario, the status quo scenario, we applied the cigarette excise taxes as stipulated in the recently approved ICE for 2023–2025 [18]. This law prescribes a nominal tax increase of 13.6% in 2023, followed by increases of 4.0% in 2024, and 3.9% in 2025, see above [18]. For this simulation scenario, we assume that the nominal excise tax increases by 4.0% annually between 2026 and 2028, and that tobacco companies fully pass the tax to consumers in the form of higher retail prices.

We compare *scenario 1* with a much more stringent scenario where the excise tax rate is increased by 30% over and above the expected annual inflationary and income increases, i.e., 36% in 2026, 46.1% in 2027, and 45.1% in 2028. We acknowledge that this percentage is arbitrary and substantial, but we believe that it is economically and politically feasible, in light of the current extremely low tax burden. We refer to this simulation as *scenario 2*. We also assume full tax pass-through for *scenario 2*.

In highly concentrated markets, industries are likely to over-shift the excise tax, i.e., they increase the retail price by more than the increase in the excise tax [43,44]. This means that the industry increases the net-of-tax price at the same time that the excise tax increases. British American Tobacco (BAT) accounts for over 90% of the market in Mozambique, suggesting that the market is fairly concentrated [45]. Premium and most-sold price categories are often subject to more over-shifting than economy segments [46–48].

Due to the uncertainty about the tobacco industry’s response to the excise tax increases, we created another simulation scenario (*scenario 3*) where the excise tax is over-shifted. In *scenario 3* the tax is assumed to increase by the same percentage as in *scenario* 2. Based on the international experience, we assumed an over-shifting parameter of 1.5 for imported, 1.3 for the most-sold brands and 1.0 (perfect pass-through) for economy brands [46,48]. This implies that the industry would increase the retail price by 1.5 times the amount of the tax increase for imported brands, by 1.3 times the amount of the tax increase for the most-sold brands, and fully pass on the excise tax to consumers of the economy brands [38]. Evidence from Bangladesh, Mauritius, Malawi and Nigeria suggests that the industry typically over-shifts the tax for premium brands and fully passes on or even under-shifts the tax for economy brands [46,49,50]. The tax shifting parameters are in line with evidence from the region on the tobacco industry’s response to excise-tax increases [46,51].

As illicit cigarettes are not subject to tax, they are not subject to over- or under-shifting. However, evidence indicates that their prices tend to rise in response to increases in the price of legal cigarettes [52,53]. Legal economy brands are probably the most “comparable” to illicit cigarettes, i.e., the closest competitor to illicit cigarettes. In the baseline, the illicit cigarettes sell at half the price of legal economy brands. We assume that the illegal cigarettes continue to sell at half the price of the economy brands after the tax changes in all scenarios.

For all three scenarios, we simulated five outcomes: cigarette prices, cigarette-tax shares of the retail prices, cigarette consumption, cigarette-tax revenue (excise tax revenue and total tax revenue, which includes all other taxes), and the adult smoking-prevalence rate. The computational procedures for these five outcomes are described in the S1 Appendix.

Unlike previous studies that primarily estimate price elasticities or assess historical tax changes in Africa, this study introduces a forward-looking simulation model tailored to Mozambique’s market structure and economic projections. By incorporating cross-price elasticities, income effects, and industry over-shifting behaviour, the TETSiM provides nuanced insights into how different tax scenarios affect consumption, prevalence, and revenue. This approach moves beyond descriptive analysis and offers actionable projections for policymakers.

### Sensitivity analysis

In the absence of country-specific demand elasticities, we used estimates from the available literature [16,27,36,54]. However, for each scenario, we adjusted the price and income elasticities by up to +/- 0.2 units above and below the elasticities listed in Table 1. For the cross-price elasticities we adjusted the elasticities by between 0.05 below and 0.1 units above the cross-price elasticities listed in Table 1. The outcomes of these variations in demand elasticities are depicted as error bars in Figs 3 and 4, and S2 Fig, illustrating the effects of varying the demand elasticities on cigarette consumption, cigarette-tax revenue, and cigarette-smoking prevalence.

**Fig 2:**
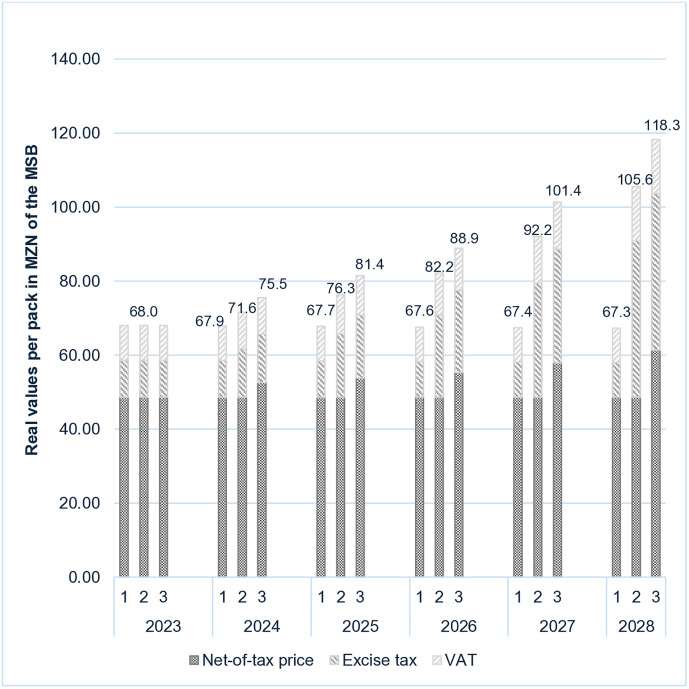
Real retail selling price of the most-sold brand (MSB). 1 = scenario 1, 2 = scenario 2, 3 = scenario 3.

**Fig 3:**
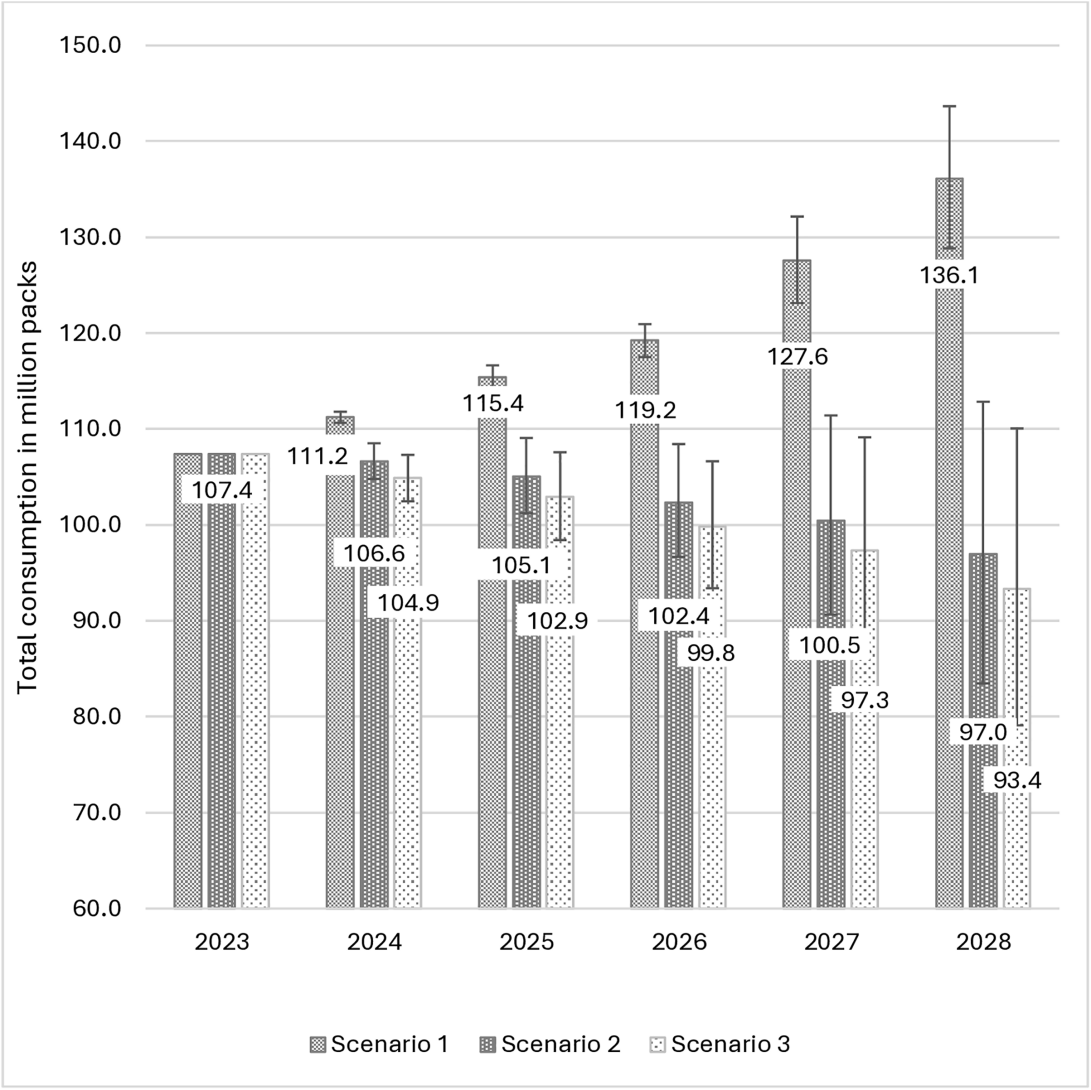
Total simulated cigarette consumption in million packs. The error bars reflect the estimates when price and income elasticities are adjusted by +/- 0.2 units above and below the elasticities listed in Table 1, and the cross-price elasticities are adjusted by between 0.05 below and 0.1 units above the cross-price elasticities listed in Table.

**Fig 4:**
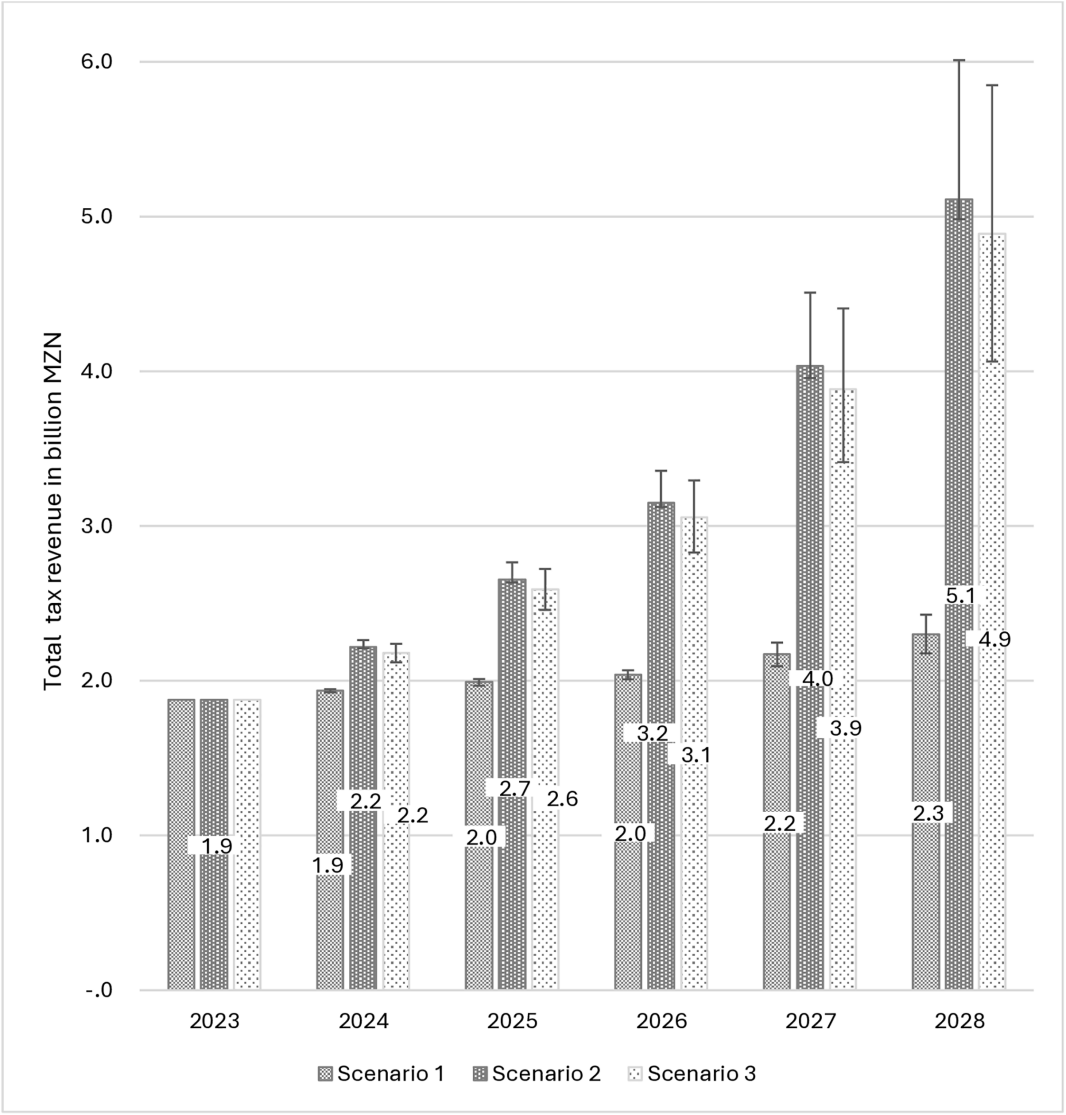
Total simulated real tax revenue in constant 2023 prices. The error bars reflect the estimates when price and income elasticities are adjusted by +/- 0.2 units above and below the elasticities listed in Table 1, and the cross-price elasticities are adjusted by between 0.05 below and 0.1 units above the cross-price elasticities listed in Table 1.

## Results

### Prices

The predicted impacts of the tax changes on the real retail price of the most-sold brand in the three scenarios are shown in Fig 2. *Scenario 1* results in the real retail price (expressed in constant 2023 prices) of the most-sold brand decreasing from MZN 68.00 in 2023 to MZN 67.27 by 2028. The real price per pack is also predicted to decrease over time for economy (1.8%) and imported cigarettes (0.5%) under scenario 1. For *scenario 2*, the real retail selling price of the most-sold brand is simulated to increase from MZN 68.00 to MZN 105.58 during the same period, a 55% increase, while for *scenario* 3, the real price increases to MZN 118.30 by 2028, equivalent to a 74% increase. For economy and imported cigarettes, the real prices per pack are also predicted to increase under scenarios 2 (94% and 24%, respectively), and 3 (94% and 38% respectively).

Our results show that in *scenario 1*, the excise tax share is expected to decrease from 14.7% in 2023 to 13.9% of the retail selling price of the most-sold brand by 2028, and the total tax share will decrease from 28.5% to 27.7% of the retail selling price during the same period. The simulation results for *scenario 2* suggest that the excise tax share of the retail price of the most-sold brand would increase from 14.7% in 2023 to 40.2% by 2028. The total tax share of the most-sold brand would increase from 28.5% in 2023 to 53.9% by 2028, which is similar to the current SADC average. For *scenario 3*, the excise tax increases from 14.7% to 35.8% and the total tax share of the retail price increases from 28.5% to 48.1% during the same period (S1 Fig).

### Consumption and tax revenue

Under *scenario 1* cigarette consumption is likely to increase between 2023 and 2028, from 107.36 million to 136.11 million packs. The increase in consumption is attributed to an increase in the population (14.52 million packs), an increase in per capita income (12.84 million packs) and a modest decrease in the price of cigarettes (1.39 million packs). Under *scenario 2*, cigarette consumption is likely to decrease by 9.67%, from 107.36 million to 96.98 million packs by 2028. The decrease in consumption is attributed to a substantial increase in the price of cigarettes, despite an increase in the population (13.05 million packs), and an increase in per capita income (10.65 million packs). The price increase would result in a decline in cigarette demand (34.09 million packs). For *scenario 3*, the total consumption is estimated to fall by 13.05% from 107.36 million to 93.36 million packs by 2028. This cumulative decrease is attributed to population growth (12.81 million packs), income growth (10.41 million packs), and a substantial price increase (37.22 million packs). The annual population and income effect on cigarette demand is dependent on the prices in the previous period, under each scenario. Despite the drop in cigarette consumption under *scenarios 2* and *3*, real total cigarette tax revenue (in constant 2023 prices) is expected to increase from MZN 1.87 billion to MZN 5.11 billion and MZN 4.89 billion, respectively, between 2023 and 2028. However under *scenario 1*, real cigarette tax revenue would only increase from MZN 1.87 billion to MZN 2.30 billion during the same period. These results are illustrated in Figs 3 and 4.

### Smoking prevalence

Our results indicate that the adult cigarette-smoking prevalence rate will drop marginally, from 9.80% to 9.77% between 2023 and 2028 under *scenario 1*. However, under *scenarios 2 and 3*, the adult cigarette-smoking prevalence rate is expected to decline from 9.80% to 8.23% and 8.08%, respectively (S2 Fig).

The empirical literature indicates that approximately half of the impact of a reduction in cigarette consumption is reflected in a decrease in the number of consumers, whereas the other half is reflected in a decrease in smoking intensity (i.e., in the average number of cigarettes smoked by remaining cigarette consumers) [15,16]. Tables 3 and 4 summarise the simulated effects of the cigarette taxes under the three scenarios, for legal cigarettes.

**Table 3:** Simulated number of legal cigarette consumers after tax change in thousands.

	2023 (Baseline)	2024	2025	2026	2027	2028
**Scenario 1**						
Imported	34.1	34.7 (34.6, 34.8)	35.3 (35.0, 35.4)	35.8 (35.4, 36.0)	36.8 (35.9, 37.5)	37.8 (36.49, 38.88)
Most-sold	869.9	885.5 (883.2, 887.8)	901.9 (896.9, 906.7)	916.6 (909.9, 923.0)	948.6 (931.5, 965.6)	979.7 (952.7, 1007.1)
Economy	716.4	730.4 (728.4, 732.4)	745.3 (741.2, 749.9)	758.7 (753.0, 764.9)	790.0 (775.5, 805.2)	820.6 (797.5, 844.8)
**Scenario 2**						
Imported	34.1	34.0 (32.8, 34.6)	33.6 (31.0, 35.1)	33.1 (28.7, 35.5)	32.3 (24.7 36.6)	31.0 (18.6, 37.5)
Most-sold	869.9	870.2 (862.8, 877.3)	867.2 (851.5, 882.4)	857.1 (831.2, 881.6)	853.7 (809.4, 879.9)	843.0 (780.5, 907.6)
Economy	716.4	709.6 (701.1, 718.9)	699.3 (681.7, 719.3)	686.4 (658.8, 719.3)	675.5 (630.1, 730.5)	658.9 (596.6, 736.6)
**Scenario 3**						
Imported	34.1	32.8 (31.2, 34.4)	32.1 (29.5, 34.5)	31.2 (27.2, 35.2)	29.8 (23.6, 36.1)	27.8 (18.8, 36.9)
Most-sold	869.9	855.4 (842.3, 866.9)	849.2 (826.2, 869.8)	835.3 (800.0, 871.9)	827.3 (771.4, 886.6)	812.0 (736.2, 897.8)
Economy	716.4	711.4 (703.8, 722.7)	701.6 (686.1, 724.0)	689.4 (664.3, 725.7)	679.0 (642.0, 738.3)	662.9 (631.4, 745.9)

**Table 4:** Simulated cigarettes smoked per smoker per week.

	2023 (Baseline)	2024	2025	2026	2027	2028
**Scenario 1**						
Imported	0.5	0.5 (0.5, 0.5)	0.5 (0.5, 0.5)	0.5 (0.5, 0.5)	0.5 (0.5, 0.5)	0.5 (0.5, 0.5)
Most-sold	12.3	12.6 (12.5, 12.6)	12.8 (12.7, 12.9)	13.0 (12.9, 13.1)	13.4 (13.2, 13.6)	13.8 (13.5, 14.2)
Economy	10.2	10.4 (10.4, 10.4)	10.6 (10.5, 10.7)	10.8 (10.7, 10.9)	11.3 (11.1, 11.5)	11.8 (11.5, 12.1)
**Scenario 2**						
Imported	0.5	0.5 (0.5, 0.5)	0.5 (0.5, 0.4)	0.5 (0.3, 0.5)	0.5 (0.3, 0.6)	0.5 (0.1, 0.6)
Most-sold	12.3	12.4 (12.3, 12.5)	12.4 (12.2, 12.6)	12.3 (11.9, 12.6)	12.3 11.6, 12.9)	12.2 (11.2, 13.1)
Economy	10.2	10.0 9.9, 10.2)	9.8 (9.5, 10.2)	9.6 (9.0, 10.2)	9.3 (8.5, 10.4)	9.0 (7.9, 10.5)
**Scenario 3**						
Imported	0.5	0.5 (0.4, 0.5)	0.5 (0.4, 0.5)	0.4 (0.3, 0.5)	0.4 (0.2, 0.5)	0.3 (0.1, 0.6)
Most-sold	12.3	12.1 (11.7, 12.3)	12.0 (11.6, 12.3)	11.8 (11.2, 12.2)	11.7 (10.7, 12.4)	11.5 (10.2, 12.5)
Economy	10.2	10.1 (10.0, 10.4)	10.0 (9.7, 10.4)	9.8 (9.3, 10.5)	9.6 (8.9, 10.7)	9.3 (8.4,10.9)

Numbers in parenthesis reflect the estimates when price and income elasticities are adjusted by +/- 0.2 units above and below the elasticities listed in Table 1, and the cross-price elasticities are adjusted by between 0.05 below and 0.1 units above the cross-price elasticities listed in Table 1.

## Discussion

Between 2003 and 2022/2023, tobacco consumption dropped from 14% to 11% for men (aged 15–49 years) and from 1.6% to 1.5% for women (aged 15–49 years) in Mozambique [55]. However, the recently approved ICE [18] is unlikely to substantially reduce cigarette use over the medium term. The proposed annual tax increases of 4% in 2024 and 2025 are not enough to counteract the effects of inflation and income growth so as to substantially reduce cigarette consumption in these two years. The TETSiM predicts that by 2028 cigarette consumption will increase, with only a marginal change in cigarette-smoking prevalence, relative to 2023. Furthermore, between 2023 and 2028 the excise tax share and the total tax share of the retail price are expected to decrease.

A strong commitment among tobacco-control stakeholders is essential to safeguard the decreases in tobacco use that have been achieved in the period 2005–2020. Cigarette-smoking prevalence in Mozambique is likely to increase if cigarettes become more affordable over time [56]. Increasing the excise tax in line with inflation and income growth is paramount to reducing the low tobacco use, and discouraging non-smokers from initiating smoking, particularly the youth.

Irrespective of how it is defined (e.g., in international dollars or as a percentage of the retail price), the excise tax in Mozambique is extremely low. The proposed excise-tax increases in *scenarios* 2 and 3 will make the tax burden more comparable to the SADC average [15].

The tobacco industry and other detractors are likely to argue that such consistently large increases in the excise tax are politically and economically infeasible. Such an argument is largely self-serving and is a common tactic used by the industry to prevent tax increases [57]. A number of countries have implemented very sharp increases in the tobacco excise tax over a number of years, often from a much higher base tax than that of Mozambique. For example, Cabo Verde increased the specific component of their tobacco excise tax from CVE 20 per pack in 2020 to CVE 40 in 2021, to CVE 70 in 2022, to CVE 90 in 2023, and to CVE 120 per pack in 2024, an average annual increase of 59% between 2020 and 2024 [58,59]. Like Mozambique, the tobacco market in Cabo Verde is fairly concentrated, with the market dominated by a single producer, *Sociedade Caboverdiana de Tabacos* [60]. Furthermore, Cabo Verde’s smoking prevalence of 9.6% in 2019 is similar to Mozambique’s smoking prevalence of 9.8% in 2020 [60,61].

The experience of the Philippines is also instructive. Before 2012, the Philippines had the cheapest cigarettes in south-east Asia and one of the lowest tax shares in the region [62]. The Sin Tax Reform Act 10351 (RA10351) of 2012 abolished the tiered tax system, and resulted in the excise tax increasing by an average of 63.7% per year between 2011 and 2017. As a result, the nominal price of the most-sold cigarettes increased by 124.4% between 2012 and 2017 [62]. The excise tax revenue grew by 231.2% between 2012 and 2017 [62]. Tobacco-use prevalence decreased from 29.7% in 2009 to 19.5% in 2021 [63].

In the mid-1990s, South Africa adopted a stringent tobacco-control policy, anchored by large increases in the excise tax. Between 1994 and 2001, the inflation-adjusted tax was increased by an average annual rate of 15.8% [64]. In this seven-year period, the industry increased the real net-of-tax price by an average of 6.6% per year, which meant that the average real retail price was increasing at a rate of 9.6% each year [64]. This increased both tax revenue and the industry’s profits despite a sharp decrease in the quantity of sales.

Mozambique’s excise tax is particularly low, which makes it easy to increase the tax by a relatively large percentage over an extended number of years. Countries with a high tax burden at the outset have shown that they can successfully increase the excise tax over many years. For example, in 2010, Australia increased its already-high cigarette excise tax by 25%, followed by eight subsequent annual increases of 12.5% [65].

In all countries, the sharp increases in the excise tax resulted in declines in cigarette consumption [62,65,66]. The cigarette market can absorb higher taxes without significant harm to the tobacco industry. In fact, the tobacco companies have been able to increase their short-term profits by over-shifting the excise tax. In Mozambique the conditions for over-shifting are ideal. Mozambique has a large tobacco company, BAT, with over 90% of the market share [45]. Sheikh, et al. found that cigarette excise tax over-shifting is common, and has been done in many countries [43]. International evidence suggests that the industry typically over-shifts the tax for premium brands and fully passes on or even under-shifts the tax for economy brands [46,49,50].

Our findings have broader relevance for LMICs with low tobacco-tax shares and concentrated markets. Countries such as Malawi, Zambia, and Angola face similar challenges of affordability and industry dominance [49,67,68]. Applying a model like TETSiM can help these countries design tax policies that anticipate industry pricing strategies and consumer switching behaviour. Moreover, the evidence from Mozambique reinforces global best practice: large, sustained excise-tax increases are feasible and effective even in markets with historically low tax burdens.

Some consumers react to tobacco-price increases by switching from more expensive products or brands to cheaper substitutes, thus diminishing the overall impact of the tobacco-tax increase on the overall tobacco demand [39]. However, this does not eliminate the impact of tobacco-tax increases. Mozambique should ensure that the tobacco-tax share of the retail price for all tobacco products, not just cigarettes, is designed such that they minimize the incentive for users to switch from less affordable tobacco products or brands to more affordable substitutes. In particular, the excise tax on other tobacco products should be increased in line with the increase in the excise tax on cigarettes.

### Limitations

Ideally, country-specific estimates of the model input parameters should be used for the simulation exercise. To the best of our knowledge, there are no studies on price, income, and cross-price elasticities for cigarettes in Mozambique, with the exception of one study by Ho et al. on price elasticities for the most-sold brand, which we use in our analysis [27]. This study therefore relies on international evidence from developing countries rather than Mozambique-specific estimates for these elasticities. To address any biases arising from the demand elasticities used in our model, we simulated each scenario with price and income elasticities that vary by +/- 0.2 units, and cross-price elasticities that vary by between 0.05 and 0.1 units from the estimates presented in Table 1.

The model allows some smokers to switch from the legal market to the illicit market when faced with higher prices, despite the fact that there is no strong evidence that tax increases will necessarily result in an increase in illicit trade [69–71]. Evidence suggests that illicit trade is currently not a problem in Mozambique [22]. It may be possible that the industry and other players will aim to undermine the large tax increases by stimulating illicit cigarette trade. Should this happen, the revenue impact of the tax increase will be undermined.

## Conclusion

This study is critical because Mozambique’s current excise tax share (14.7%) is among the lowest in Southern Africa, making cigarettes increasingly affordable. Without substantial tax increases, smoking prevalence will stagnate, undermining progress on NCD prevention and on meeting the Sustainable Development Goals. The sharp excise-tax increases would substantially increase cigarette prices. Should the cigarette excise tax in Mozambique be increased by 30% above the sum of the per-capita GDP growth and inflation rates each year for five years, the tax/price ratio would be similar to that of the SADC average. Our projections demonstrate that aligning tax policy with inflation and income growth, and adding a significant real increase, can simultaneously reduce smoking prevalence and triple tax revenue, creating a win-win for public health and fiscal sustainability.

We therefore recommend that the Mozambican government adjust the cigarette excise tax annually by at least 30% over and above the sum of the per-capita GDP growth rate and inflation rate. This will result in an increase in the excise tax share and the total tax share of the retail price. It will make cigarettes substantially less affordable. Furthermore, it will reduce cigarette consumption and smoking prevalence, but increase cigarette tax revenue. It will be good for both public health and the fiscus.

## Supporting information


S1 Appendix.
TETSiM methodology. (DOCX)

S1 Fig Simulated cigarette tax share of retail price of the most sold brand (MSB). (TIF)

S2 Fig Simulated cigarette smoking prevalence rate. (TIF)

S1 Model.
Simulation scenario 1. (XLSX)

S2 Model.
Simulation scenario 2. (XLSX)

S3 Model.
Simulation scenario 3. (XLSX)
