# Supplementary material for: Making cigarette taxes more effective in Mozambique: A simulation analysis using the Tobacco Excise Tax Simulation Model (TETSiM)
Source: PLoS One. 2026 Feb 2;21(2):e0341079. doi: 10.1371/journal.pone.0341079 (PMC12863564; doi:10.1371/journal.pone.0341079)
Supplement: S1 Appendix. — (DOCX) [file pone.0341079.s006.docx]

**Making cigarette taxes more effective in Mozambique: A simulation analysis using the Tobacco Excise Tax Simulation Model (TETSiM) – S1 Appendix**

#### Simulated cigarette prices

The retail price of domestically-produced cigarettes can be decomposed into the net-of-tax (NOT) price, the excise tax and the VAT amount. The small volumes of imported cigarettes are also subject to an import duty, which is levied at a rate of 25% on the cost, insurance, and freight (CIF) value of the product. For the most-sold brand, the retail price (MZN 68.00) in the baseline (2023) decomposes into a NOT value of MZN 48.62, an excise tax of MZN 10.00, and VAT of MZN 9.38, which is levied at a rate of 16% on the sum of the NOT price, the excise tax and all other taxes.

For the subsequent years, we assume that the nominal CIF values (for imported cigarettes) and NOT prices (for domestically produced cigarettes) increase by the inflation rate. For *scenario 1* the nominal excise tax increases in line with the Tax on Specific Consumption Law revisions for 2023 to 2025, and by an assumed 4% in 2026 to 2028, which is consistent with the annual increases between 2023 and 2025. For *scenarios 2* and *3*, the excise tax increases by the sum of the predicted annual per-capita GDP growth rate, inflation rate, and 30%.

# **Methods**

## **Cigarette tax shares**

The excise tax shares were derived by dividing the excise tax amount by the retail selling price. For the most-sold brand, this equated to 14.7% (10.00/68.00) in 2023. For the total tax shares, we divided the sum of the excise tax and VAT by the retail selling price. This equated to 28.5% [(10.00+9.38/68.00] in 2023 for the most-sold brand.

### Simulated cigarette consumption

According to the TETSiM, the growth in aggregate cigarette consumption is determined by three magnitudes: changes in the real price of the product, changes in per-capita income (growth), and changes in the population. The own-price elasticity quantifies the impact of the price changes, broken down by market segment, on cigarette consumption. Similarly, the income elasticity quantifies the impact of income changes (proxied by the real per-capita GDP growth rate), on cigarette consumption, also broken down by market segment. The change in the population impacts aggregate consumption, but has no impact on per capita consumption. Aggregate cigarette consumption in period *t* is equal to aggregate cigarette consumption in period *t-1*, adjusted for the changes in price, per capita income and population. Eq 1 summarises the computational procedure.

${SimCons}_{bt}={Cons}_{bt-1}*\left[ \frac{\left( 1+\varepsilon_{pb}\left( p_{bt}-p_{bt-1} \right) \right)}{\left( p_{bt}+p_{bt-1} \right)}*\frac{\left( p_{bt}+p_{bt-1} \right)}{1-\varepsilon_{pb}\left( p_{bt}-p_{bt-1} \right)} \right]*\left( 1+{pop}_{t} \right)\left( 1+{inc}_{t}*\varepsilon_{incb} \right) ($1)

${SimCons}_{bt}$ is simulated cigarette consumption at year *t* for market segment *b*. ${Cons}_{bt-1}$is the cigarette consumption in market segment *b* in the previous year. $\varepsilon_{pb}$is the own-price elasticity for market segment *b*. $p_{bt}$and $p_{bt-1}$ are the prices per pack for market segment *b* at year *t* and year *t–1*, respectively. ${pop}_{t}$ is the population growth rate in year *t* and ${inc}_{t}$ is the real per capita GDP growth rate in year *t.* $\varepsilon_{incb}$ is the income elasticity for market segment *b*. The expression in square parentheses is derived from the arc elasticity formula for the price elasticity of demand, where the new quantity becomes the subject of the formula; see C. Van Walbeek [1].

Other than reducing their consumption because of the price increase, it is possible that some consumers will switch from more expensive cigarettes to cheaper cigarettes. We took account of this substitution effect using cross-price elasticities. We simulated the substitution effect of cigarette demand from a higher-priced market segment to a lower-priced market segment, using Eq 2:

${\Delta SimCons}_{sbt}=\frac{p_{at}-p_{at-1}}{0.5(p_{at}+p_{at-1})}*\varepsilon_{bx}*{SimCons}_{bt}$ (2)

where ${\Delta SimCons}_{sbt}$ is the simulated change in consumption attributed to consumers switching from a higher-priced market segment *a* to a lower-priced market segment *b.* $p_{at}$ and $p_{at-1}$ are the prices for the higher-priced market segment *a* in year *t* and year *t–1,* respectively. $\varepsilon_{bx}$ is the cross-price elasticity, defined as the percentage change in consumption for the lower-priced market segment *b* attributed to a 1% price change in the higher-priced market segment *a*. ${SimCons}_{bt}$ is the simulated cigarette consumption in year *t* for market segment *b*, derived from Eq 1.

The increase in consumption in the lower-priced market segment *b* is subtracted from the consumption in the higher-priced market segment *a*. The net effect of the substitution effects on aggregate consumption is zero.

To estimate total cigarette consumption (by market segment) after the tax change (and accounting for income and population growth), we added the simulated cigarette demand (Eq 1), and the simulated change in cigarette demand attributed to the substitution effect (Eq 2); (Eq 3).

$${TotalSimCons}_{bt}={SimCons}_{bt}+{\Delta SimCons}_{sbt} (3)$$

Total simulated cigarette consumption is the sum of the consumption in each of the B market segments, (Eq 4):

${TotalSimCons}_{t}= \sum_{b}^{B} {SimCons}_{bt}$ (4)

#### Simulated tax revenue

To calculate the simulated tax revenue, we multiplied the simulated quantities by the cigarette taxes, for each market segment. For *scenario 1*, we used the excise taxes stipulated in the Excise Tax on Specific Products Law (ICE). For *scenarios 2* and *3*, we used the proposed excise taxes and the prevailing VAT*.* We estimated the simulated tax revenue as per Eq 5.

${Simtaxrev}_{it}=\sum_{b=1}^{B} {tax}_{it}*{TotalSimCons}_{bt}$ (5)

Where ${Simtaxrev}_{it}$ is the simulated tax revenue for all market segments for tax *i* (i.e. excise tax, and VAT) in year *t*. ${tax}_{it}$ is the tax amount per pack (for tax *i*) in year *t*. ${TotalSimCons}_{bt}$ is the total simulated cigarette consumption in year *t*, derived from Eq 3.

### Simulated cigarette prevalence rate

The cigarette prevalence rate is defined as the number of cigarette consumers divided by the adult population (aged 15 and above). The number of cigarette consumers is expected to change as cigarette consumption changes. The empirical literature indicates that approximately half of the impact of a reduction in cigarette consumption is reflected in a decrease in the number of cigarette consumers, whereas the other half is reflected in a decrease in smoking intensity (i.e., in the average number of cigarettes smoked by remaining cigarette consumers) [2, 3]. See Eq 6:

${Smokers}_{t}={Smokers}_{t-1}\left( 1+\%{\Delta TotalSimCons}_{t}*50\% \right)$ (6)

${Smokers}_{t}$ is the number of cigarette consumers in year *t*. $\%{\Delta TotalSimCons}_{t}$ is the percentage change in the total simulated cigarette consumption between year *t* and year *t–1*.

References

1. C. Van Walbeek. A Simulation Model to Predict the Fiscal and Public Health Impact of a Change in Cigarette Excise Taxes. Tobacco Control. 2010;19(1):31.10.1136/tc.2008.028779

2. International Agency for the Research on Cancer (IARC). Effectiveness of Tax and Price Policies for Tobacco Control. Iarc Handbooks of Cancer Prevention in Tobacco Control. Lyon, France.: International Agency for the Research on Cancer; 2011 [Available from: <https://web.archive.org/web/20220125151614/https://publications.iarc.fr/_publications/media/download/4018/05229a5e57f58b0bf51364dd0f3329d45c898839.pdf>.

3. WHO Framework Convention on Tobacco Control. Guidelines for Implementation of Article 6 of the Who Fctc (Price and Tax Measures to Reduce the Demand for Tobacco) Moscow, Russian Federation: WHO FCTC; 2017 [Available from: <https://fctc.who.int/publications/m/item/price-and-tax-measures-to-reduce-the-demand-for-tobacco>.
