# Supplementary figures and images for: Making cigarette taxes more effective in Mozambique: A simulation analysis using the Tobacco Excise Tax Simulation Model (TETSiM)
Source: PLoS One. 2026 Feb 2;21(2):e0341079. doi: 10.1371/journal.pone.0341079 (PMC12863564; doi:10.1371/journal.pone.0341079)

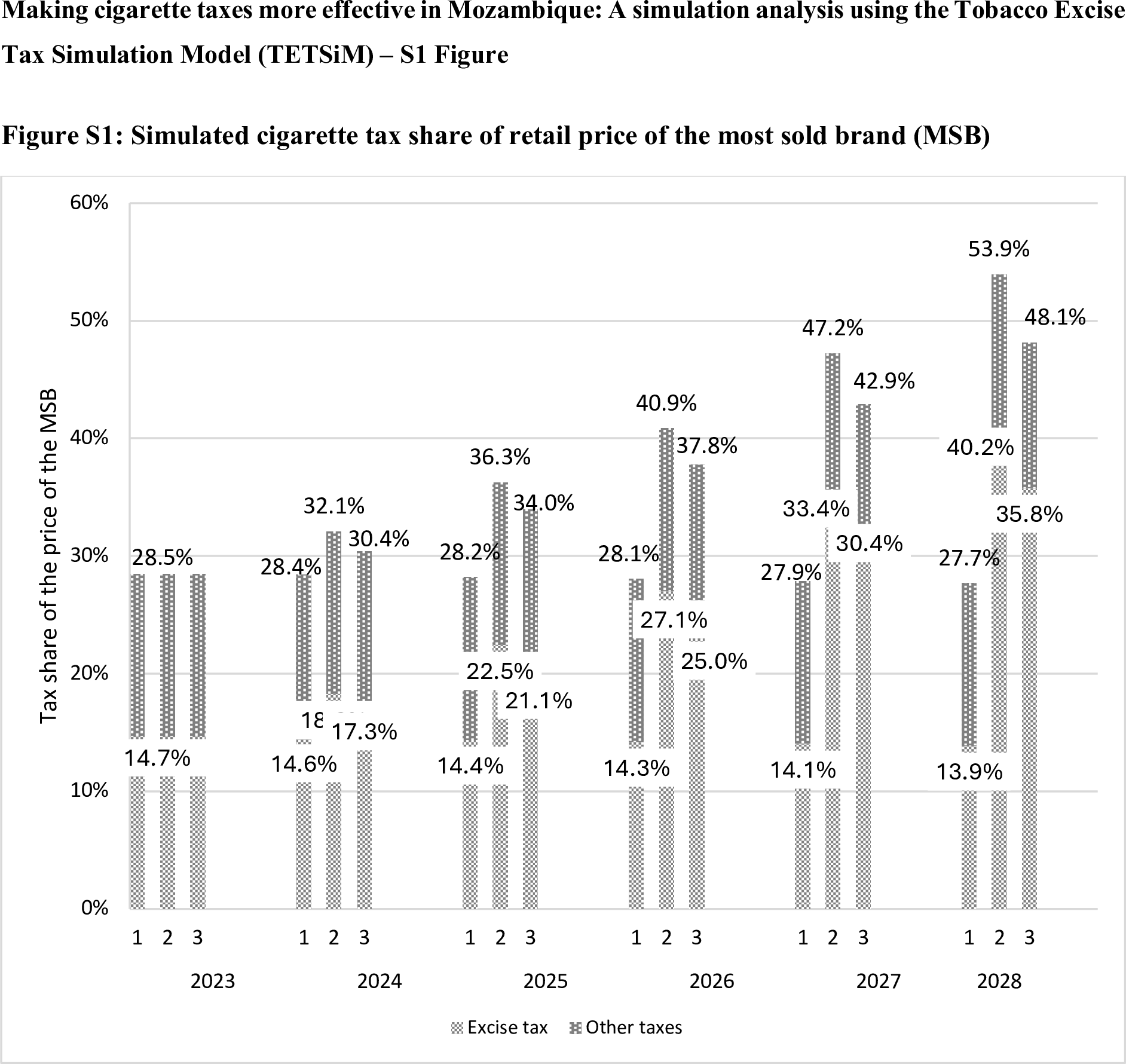

Supplement: S1 Fig — (TIF) [file pone.0341079.s001.tif]

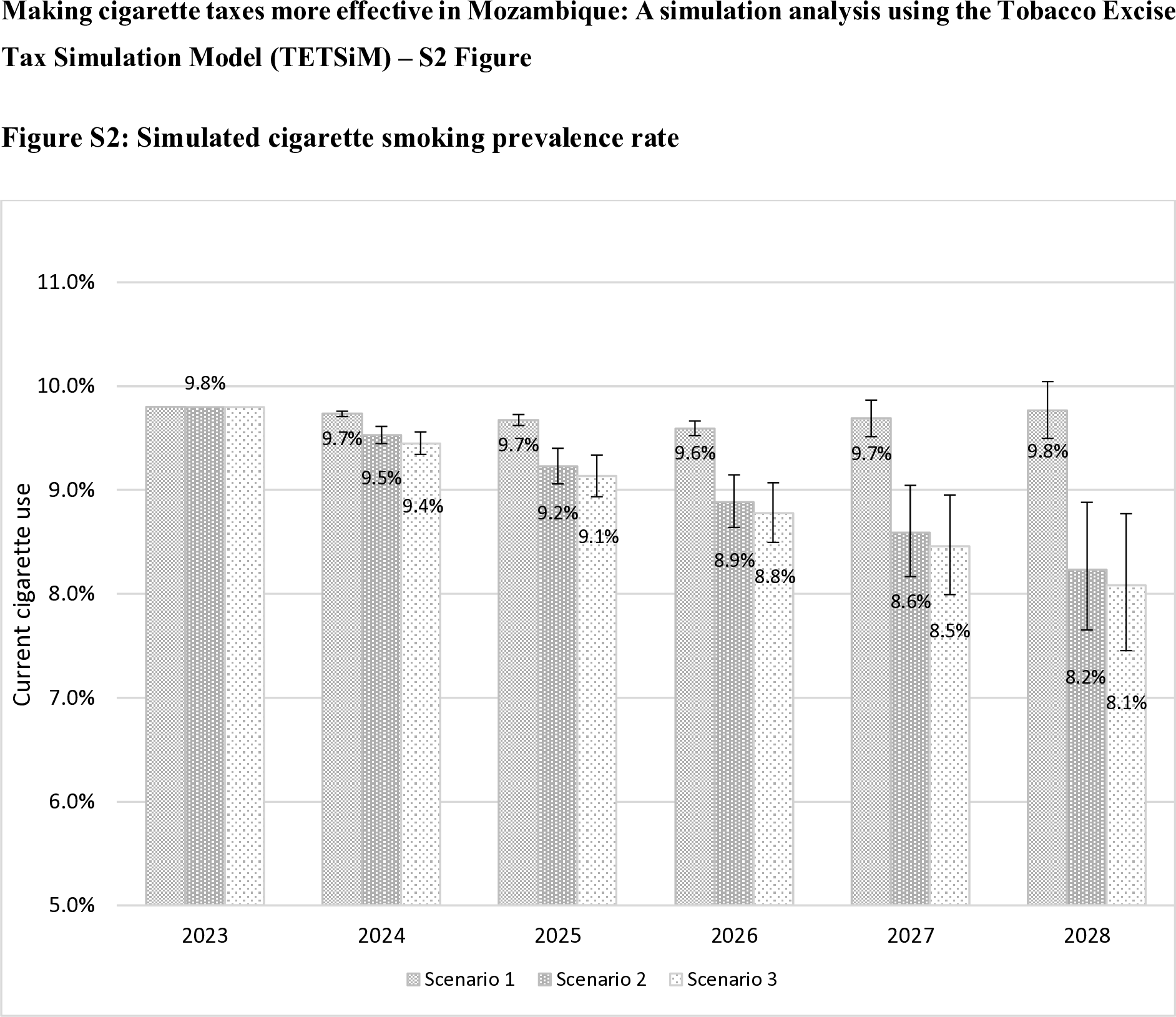

Supplement: S2 Fig — (TIF) [file pone.0341079.s002.tif]
